# The mediating effect of subject well-being between physical activity and the internet addiction of college students in China during the COVID-19 pandemic: a cross-sectional study

**DOI:** 10.3389/fpubh.2024.1368199

**Published:** 2024-04-05

**Authors:** Jinfu Wang, Xue Xu, Qinmei Wu, Chao Zhou, Guan Yang

**Affiliations:** ^1^School of Physical Education, South China University of Technology, Guangzhou, Guangdong, China; ^2^School of Finance and Economy, Guangdong Engineering Polytechnic, Guangzhou, Guangdong, China; ^3^School of Physical Education, Shandong University, Jinan, Shandong, China

**Keywords:** internet addiction, subjective well-being, physical activity, mediating effect, college students, the COVID-19 pandemic, a cross-sectional study

## Abstract

**Background:**

Internet addiction poses a significant threat to the health of college students worldwide, but physical activity, as a highly safe and effective rehabilitative measure, has shown promise for alleviating this issue nowadays. However, during the COVID-19 pandemic, the mediating processes in this association remained unclear. This study aims to explore the impact of physical activity on internet addiction among college students and the mediating role of subjective well-being.

**Methods:**

A survey was conducted on 216 eligible college students using the physical activity level scale, the internet addiction test, and the subjective well-being scale. For data analysis, independent sample t-tests, correlation analysis, hierarchical regression analysis, and mediating effect tests were in turn carried out in this work.

**Results:**

The study revealed noteworthy gender disparities in physical activity and internet addiction among college students (*β* = −0.356, *p* < 0.01; *β* = 0.140, *p* < 0.05). Compared to females, male students manifest elevated levels of physical activity and lower scores in internet addiction. Physical activity and subjective well-being exerted a significantly negative predictive influence on internet addiction (*β* = −0.162, *p* < 0.05; *β* = −0.508, *p* < 0.001). What’s more, subjective well-being assumed a crucial mediating role in the relationship between physical activity and internet addiction, with the mediating effect accounting for 72.81% of the total effect.

**Conclusion:**

This study deepens the understanding of how physical activity reduces internet addiction risk while emphasizing that enhancing subjective well-being is an effective strategy for college students to cope with Internet addiction.

## Introduction

Since the onset of the COVID-19 pandemic in late December 2019 across the world, the terrible virus has rapidly disseminated globally, significantly impacting worldwide politics, economics, and society (1). Due to the highly contagious nature of COVID-19, China has implemented a comprehensive suite of preventive and control measures, encompassing school closures, the enforcement of social distancing, and the imposition of stay-at-home directives (2). These measures confined college students to campuses, rendering the internet a vital instrument for learning and communication (3). Research posited that moderate internet exposure can prove beneficial, encompassing heightened attention levels (4) and enhanced cognitive functions and psychological adaptation skills (5). Nevertheless, it is imperative to acknowledge that excessive internet use may lead to various adverse consequences, such as internet addiction. Recent meta-analyses indicated that approximately 10.7% of Chinese college students contend with issues related to internet addiction, and this percentage was escalating (6). Cross-sectional studies suggested that Internet addiction is intricately linked, not only with musculoskeletal issues in university students (7) but also with a spectrum of mental disorders or behavioral challenges, encompassing depression, anxiety, and suicidal tendencies (8, 9). Hence, tackling internet addiction among university students and exploring improvement strategies is a pressing real-world concern.

In recent years, amidst the plethora of factors influencing individual addictive behaviors, the affirmative impacts of physical activities have attracted escalating attention and acknowledgment. A multitude of studies have elucidated the connection between physical activities and internet addiction, encompassing diverse intensities (10), durations (11), and various types of physical activities and exercise regimens (12). For instance, Fan et al. (10) discerned that engaging in moderate-intensity physical activity constitutes an efficacious strategy for ameliorating internet addiction. In comparison to both low-intensity and high-intensity physical activities, students participating in moderate-intensity physical activities reported lower levels of internet addiction. Lan et al. (11) pointed out that both long-term continuous intervention and short-term high-intensity physical activity can enhance individuals’ sense of achievement and promote the transfer of achievement feelings, consequently diminishing reliance on the internet. Liu et al. (12) posited that collective confrontational projects such as football and basketball can also effectively prevent and correct internet addiction. Furthermore, research on combined exercise and biofeedback interventions indicates that physical activity helps repair the brain structure damage of internet addiction patients and improves brain areas related to executive functions, thereby enhancing individual executive functions, self-control, and resistance to the internet (13). Similarly, physical activity could also bidirectionally regulate the conversion rate of dopamine, maintaining the effect of “immediate slowing after exercise—a gradual decrease 24 h later, “achieving the inhibition of psychological cravings and relapse behaviors related to the internet (14).

### The mediating role of subject well-being

Despite extensive discussions in previous studies on the association between individual characteristics and internet addiction, such as depression (15), anxiety (16), and loneliness (17), Nevertheless, limited research has focused on examining the link between subjective well-being and internet addiction. In these studies, subjective well-being as a crucial psychological state has not received sufficient research attention. Subjective well-being refers to an individual’s assessment and perception of their life quality, serving as a crucial comprehensive psychological indicator for measuring individual well-being (18). Abundant studies have demonstrated that subjective well-being negatively predicts an individual’s propensity for internet addiction (19–21). High subjective well-being, encompassing overall emotions and life satisfaction, is deemed a protective factor against the development of internet addiction among university students (20, 21). Moreover, the compensation theory posits that individuals, propelled by the motivation of developmental needs (internalized forms of subjective well-being), may engage in “pathological compensation” through easily achievable activities such as internet use when disruptions prevent the satisfaction of these needs (22). Conversely, if psychological needs are satisfied, the likelihood of internet addiction decreases. Hence, subjective well-being may influence Internet addiction, and augmenting subjective well-being may culminate in a reduction of internet addiction levels. Nevertheless, the current challenge lies in determining effective strategies to enhance subjective well-being.

The Basic Needs Theory postulates that the fulfillment of three needs (competence, autonomy, and relatedness) in an individual’s developmental history is conducive to global well-being and mental health (23). Previous research indicated a positive and significant association between physical activity and the three fundamental psychological needs of competence, autonomy, and relatedness (24). More precisely, if an individual’s needs for competence, autonomy, and relatedness find fulfillment in the realm of sports and physical activities, overall well-being (e.g., life satisfaction) or happiness (e.g., subjective vitality) should increase accordingly (25). Fortuitously, evidence substantiating the role of basic psychological needs delineated by Deci and Ryan (26) in advancing mental health and behavior has been manifested in the domain of sports and physical activity (27). Furthermore, several studies have evidenced a positive correlation between physical activities and subjective well-being. For instance, Yazicioglu et al. (28) indicated that, compared to those who do not participate in physical activity, engagement in physical activities can lead to higher quality of life and life satisfaction, which are components and indicators of subjective well-being. Dolan et al. (29) noted that systematic or regimented physical engagement is conducive to subjective well-being. Similarly, Herbert (30) discovered that psychophysical activities (e.g., yoga) can have an immediate adaptive impact on physical and mental health, consequently further elevating felicity. Nevertheless, it has also been found that physical activity for health or other external purposes might not increase individual subjective well-being (31). Consequently, grounded in the aforementioned theoretical and empirical evidence, it is rational to posit that there exists an inverse association between subjective well-being and internet addiction. Additionally, subjective well-being might serve as a mediating factor in the link between physical activity and internet addiction.

### The present study

In summary, the internet addiction exhibited by college students during the COVID-19 period is worrisome. Numerous studies confirm the positive influence of physical activities on individual internet addiction, along with the role of subjective well-being in alleviating these conditions. Nevertheless, in prior studies (particularly within the Chinese context), the connection between physical activities and internet addiction amid the COVID-19 period among university students, along with the underlying mechanisms, remains inadequately investigated. To fill this research gap, we conducted a thorough investigation into the association between physical activities and internet addiction among Chinese university students amid the COVID-19 pandemic.

**Figure 1:**
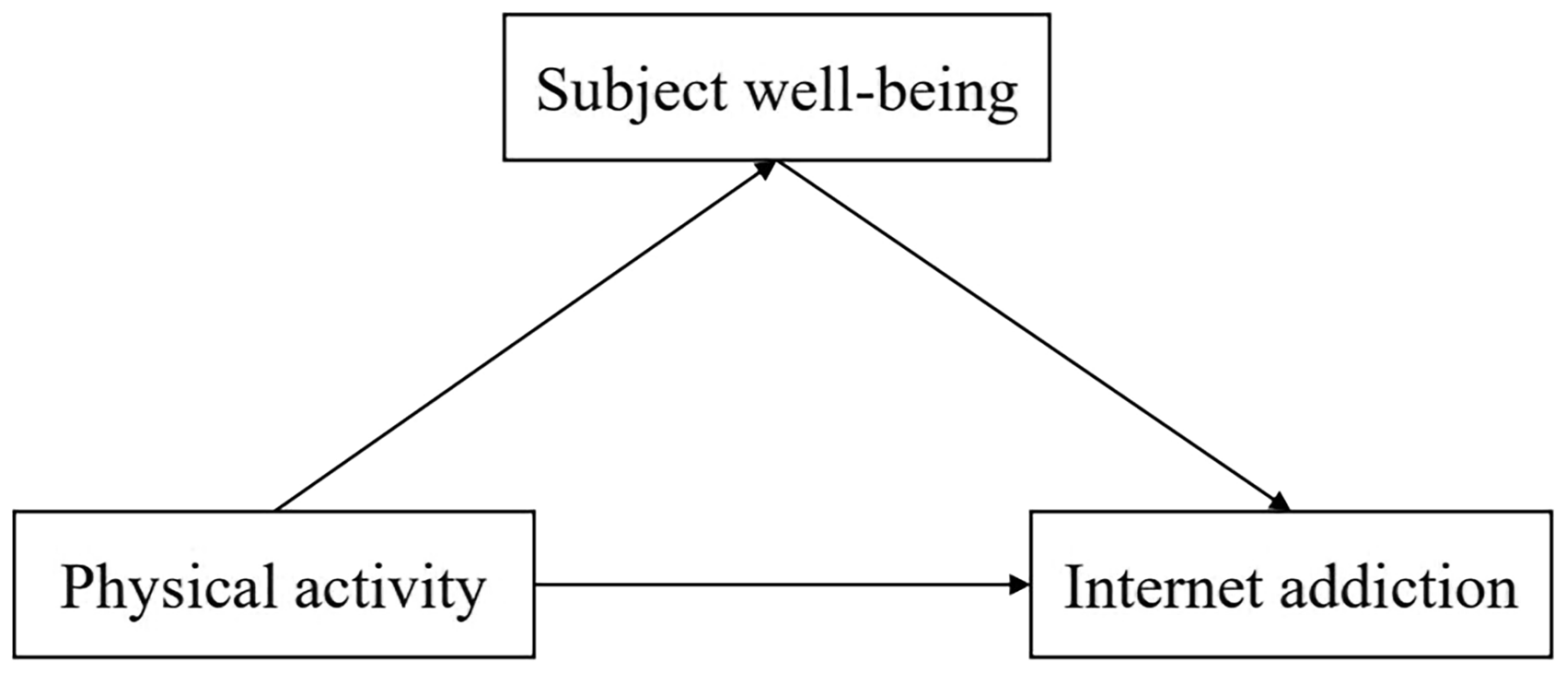
The hypothesized model in present study.

In contrast to earlier research, our study focuses on revealing the complex connections among physical activity, subjective well-being, and internet addiction. We considered the dynamic interactions between lifestyle factors and internet addiction, aiming to clarify how the interplay of physical activity and subjective well-being molds internet usage patterns in college students. The worldwide pandemic has presented unprecedented challenges, urging us to explore their potential impact on established correlations. Based on the significant correlation among these factors, we developed a simple mediation model (Figure 1) aiming to explore the mediating role of subjective well-being between college students’ physical activities and internet addiction, elucidating how physical activity influences internet addiction. Furthermore, we investigated the correlations between these factors and demographic variables such as gender and grade. We hypothesize a negative correlation between physical activity and internet addiction, with subjective well-being playing a vital mediating role between physical activity and internet addiction.

## Methods

### Procedures and participants

Given the ongoing outbreak of COVID-19 and the limitations associated with social contact, this cross-sectional study employed convenient sampling and collected data online in April 2022 at Shandong University in Jinan, Shandong Province, China. To facilitate online data collection, we opted for the Questionnaire Star questionnaire platform,^1^ and professional researchers acted as the primary experimenters. Online, researchers distributed QR codes for the survey, enabling participants to scan and complete it on their mobile phones. During participant selection, we defined inclusion and exclusion criteria to enhance study validity and representativeness. Participant inclusion criteria for participants encompass Chinese nationality, proficiency in spoken and written Chinese, being full-time university students (both undergraduates and postgraduates), and having no prior participation in similar studies. Exclusion criteria for participants comprise failure to meet inclusion criteria, irregular responses (e.g., repeated selection of the same option), and incomplete answers. Prior to questionnaire completion, professional researchers explained instructions to participants, informing them of the survey’s anonymity, confidentiality, and purpose. It was ensured that the questionnaire’s completion was based on participants’ voluntary and informed consent. Ultimately, 216 eligible college students with an average age of 20.59 years (SD = 5.78) were selected and successfully completed online questionnaires. To verify the accuracy and effectiveness of the data, researchers carefully examined the data from each completed questionnaire to ensure internal logic and consistency.

This study strictly adhered to the ethical principles of the 1964 Declaration of Helsinki and its later amendments or comparable ethical standards and was also reviewed and approved by the research ethics committee at South China University of Technology. And in the meantime, all participants provided informed consent online before officially participating in the survey.

### Measures

#### Internet addiction

The internet addiction test was utilized to assess the degree of internet addiction among college students (32). The scale comprises 20 questions, and participants rate each question on a 5-point Likert scale, ranging from 1 (almost never) to 5 (always). The total score is obtained by summing the scores for each question, with higher scores indicating more severe symptoms of internet addiction. This measure has been widely used in the Chinese university population and has shown good reliability and validity (33). In this study, the internal consistency of the Internet Addiction Scale is good, with a Cronbach’s α coefficient of 0.732.

#### Subjective well-being

Subjective well-being among college students was evaluated using the happiness scale proposed by Diener (34). The scale encompasses two dimensions: emotional and cognitive. It consists of 20 items, scored on a 5-point Likert scale. Questions 4, 5, 6, 9, 10, 11, 13, 15, 17, 18, and 20 are reverse-scored, and the total score is obtained by summing the scores for each question. This measure has been widely used in the Chinese university population and has shown good reliability and validity (35). In this study, the internal consistency of the Subjective Well-being Scale is satisfactory, with a Cronbach’s α coefficient of 0.738.

#### Physical activity

Physical activity was assessed by the Physical Activity Rating Scale (PARS-3) (36). This scale has 3 items (intensity, time, and frequency), and the amount of physical activity was calculated by the following formula: “exercise intensity × (exercise duration − 1) × exercise frequency.” The total scores for physical activity were from 0 to 100. This measure has been widely used in the Chinese university population and has shown good reliability and validity (37, 38). The internal consistency of the PARS-3 in this study was generally satisfactory, with a Cronbach’s alpha coefficient of 0.817.

#### Demographic variables

Besides the three aforementioned primary variables, all participants also provided details on several demographic features, encompassing age, gender, major, and residence.

### Statistical analysis

Statistical analysis was conducted using SPSS 19.0 software in this study. Continuous variables were presented as mean ± standard deviation (M ± SD), while categorical variables were expressed as frequencies (*n*) and percentages (%). Initially, independent sample t-tests were employed to explore potential demographic differences related to physical activity, internet addiction, and subjective well-being. Subsequently, correlation analysis investigated the interrelationships among variables. Due to the non-normal distribution of physical activity, Spearman’s rank correlation analysis was utilized to explore the relationship between physical activity and both internet addiction and subjective well-being. The association between internet addiction and subjective well-being was assessed using Pearson correlation analysis. In the third step, hierarchical regression analysis was executed to examine the mediating effect of subjective well-being between physical activity and internet addiction. Finally, the Bootstrap method (with 5,000 resamplings) was utilized to estimate the 95% confidence interval (CI) and assess the significance of the mediating effect. The direct or indirect effects were deemed significant when the CI was <0. Statistical significance was indicated by *p* < 0.05 (two-tailed).

## Results

### Common method bias analysis

The utilization of a questionnaire survey in this study introduces the possibility of common method bias. Harman’s single-factor test was employed to assess the existence of common method bias. Results from the unrotated factor analysis reveal that the variance explained by the first principal component is 29.74%, falling below the 40% critical threshold. This indicates that the study does not exhibit significant common method bias, allowing for subsequent statistical analysis.

### Sample characteristics analysis

As shown in Table 1, the sample for this study consisted of 216 college students, which comprises 118 male (54.6%) students and 98 female (45.4%) students. Of them, 47 students (21.8%) were from rural areas, and 169 students (78.2%) were from urban areas. In terms of majors, 87 students (40.3%) majored in liberal arts, and 129 students (59.7%) majored in sciences.

**Table 1:** Between-group differences in physical activity, subjective well-being, and the Internet addiction by main demographic indicators.

Variable		*N* (%)	Statistical value	Physical activity	Internet addiction	Subjective well-being
Gender	Total	216		21.31 ± 4.16	51.02 ± 17.01	61.00 ± 11.58
	Male	118 (54.6%)		28.67 ± 6.24	48.80 ± 15.33	66.00 ± 10.96
	Female	98 (45.4%)		12.45 ± 3.69	53.69 ± 18.58	66.00 ± 12.35
			*t*	5.199^***^	−2.119^*^	0.000
			Cohen’s *d*	0.731	0.287	0.000
Major	Liberal Arts	87 (40.3%)		21.63 ± 4.59	51.65 ± 15.69	66.94 ± 11.86
	Science	129 (59.7%)		21.10 ± 3.97	50.59 ± 17.91	65.36 ± 11.40
			*t*	0.156	0.447	0.982
			Cohen’s *d*	0.021	0.062	0.135
Residence	Rural	47 (21.8%)		21.27 ± 5.70	48.27 ± 14.74	65.63 ± 10.95
	Urban	169 (78.2%)		21.33 ± 3.80	51.78 ± 17.56	66.10 ± 11.78
			*t*	−0.014	−1.252	−0.241
			Cohen’s *d*	0.002	0.216	0.041

^*^p < 0.05; ^***^p < 0.001.

Independent sample t-test analysis indicated a significant difference in the physical activity levels between male and female students, with males showing higher levels [*t*_(216)_ = 5.199, *p* < 0.001, *d* = 0.731]. Conversely, the level of internet addiction was found to be higher in female students compared to male students [*t*_(216)_ = −2.119, *p* < 0.05, *d* = 0.287]. No significant variations were observed in physical activity levels, internet addiction scores, or subjective well-being scores based on factors such as major and place of residence.

### Preliminary correlation analyses

As shown in Table 2, the correlation coefficients of physical activity, internet addiction, and subjective well-being were all statistically significant. Physical activity was negatively correlated with internet addiction (*r* = −0.226, *p* < 0.05) but positively correlated with subjective well-being (*r* = 0.207, *p* < 0.05), with both correlations being relatively low. Internet addiction was negatively correlated with subjective well-being (*r* = −0.514, *p* < 0.01), showing a relatively high correlation.

**Table 2:** Correlation analysis of three main variables.

Variable	Physical activity	Internet addiction	Subjective well-being
Physical activity	—		
Internet addiction	−0.226^*^	—	
Subjective well-being	0.207^*^	−0.514^**^	—

^*^p < 0.05; ^**^p < 0.01.

### Hierarchical regression analysis

Table 3 presented the coefficients of hierarchical regression analysis for the relationship between physical activity, subjective well-being, and internet addiction. The results indicated that, in Model 2, physical activity had a significantly positive impact on subjective well-being (*β* = 0.232, *p* < 0.01). In Model 3, gender exhibited a positive and significant prediction for internet addiction (*β* = 0.140, *p* < 0.05), accounting for 2.6% of the variance in internet addiction. After controlling for variables such as gender, college, and residence, Model 4 revealed that physical activity significantly negatively influenced internet addiction (*β* = −0.162, *p* < 0.05), with an explanatory increase of 2.3% (*ΔF* = 5.096, *p* < 0.05). Finally, introducing subjective well-being into Model 5 revealed a significant negative impact of subjective well-being on internet addiction. The explanatory power increased by 24.5% (*ΔF* = 72.90, *p* < 0.001). Simultaneously, the negative impact of physical activity on internet addiction no longer held statistical significance (*p* > 0.05), suggesting a potential mediating effect of subjective well-being between physical activity and internet addiction.

**Table 3:** Results of hierarchical regression analysis.

Variable	Subjective well-being		Internet addiction		
	Model 1	Model 2	Model 3	Model 4	Model 5
Constant terms	68.59	62.76	38.09	44.07	90.94
**Control variables**					
Gender	−0.019	0.065	0.140^*^	0.081	0.114
Major	−0.071	−0.047	0.009	−0.008	−0.032
Residence	0.015	0.009	0.075	0.079	0.084
**Independent variable**					
Physical activity		0.232^**^		−0.162^*^	−0.044
**Mediation variable**					
Subjective well-being					−0.508^***^
*R* ^2^	0.005	0.052	0.026	0.049	0.294
Δ*R*^2^	0.005	0.047	0.026	0.023	0.245
F	0.356	2.903	1.902	2.728^*^	17.50^***^
Δ*F*		10.49^**^		5.096^*^	72.90^***^

^*^p < 0.05; ^**^p < 0.01; ^***^p < 0.001.

### Mediating effect test

To further verify the mediating role of subjective well-being between physical activity and internet addiction, this study employed the bootstrap method based on the above research conclusions. The results (Table 4) indicated that the confidence interval [−0.146, −0.027] of the indirect path from physical activity → subjective well-being → internet addiction did not include 0. This signified the significant mediating effect of subjective well-being, with a mediating effect size of 0.083, accounting for 72.81% of the total effect. After controlling for mediating variables, the direct path from physical activity → internet addiction was not significant (95% confidence interval included 0). This implied that subjective well-being played a complete mediating role between physical activity and internet addiction, reinforcing the findings of this study.

**Table 4:** Bootstrap analysis of the mediating effect test on subjective well-being.

Paths	Effect	Boot SE	Bias-corrected 95% CI		Effect size ratio
			Lower limit	Upper limit	
Total effect	−0.114	0.051	−0.213	−0.014	100%
PA → SWB → IA	−0.083	0.030	−0.146	−0.027	72.81%
PA → IA	−0.031	0.045	−0.119	0.057	27.19%

PA, Physical activity; IA, Internet addiction; SWB, Subject well-being; Demographic variables are controlled as covariates.

## Discussion

The present study aims to examine the underlying relationship between physical activity and internet addiction among a sample of Chinese college students and also to disclose the mediating role of subjective well-being in this relationship as well as the associations of these factors with several demographic variables, with a view to providing more in-depth theoretical insights into the role of physical activity in ameliorating internet-addictive behaviors for college students and relevant groups in China or even all over the world.

Consistent with most of the earlier research (39), our study found significant gender differences in internet addiction. In contrast to their male counterparts, female students attain higher scores on the internet addiction scale, signifying an elevated vulnerability to online addiction. Gender disparities in the brain regions related to executive control can explain why female students exhibit higher levels of internet addiction compared to male students. For instance, Wang et al. (40) reported that, in comparison to males, females manifest reduced cortical thickness in the bilateral anterior cingulate cortex, linked to heightened motivation. Motivation, serving as a motivational state indicative of the endorsement of gaming-related stimuli, has been established as a pivotal facet of addiction (40–42). Put differently, the aberrant development of the anterior cingulate cortex in females might foster a more robust motivational impetus, potentially culminating in heightened addictive behavior (40). Moreover, within this study, the levels of physical activity among male students were higher than those of their female counterparts. This outcome was not surprising, and a reasonable explanation is that, compared with females, male students have higher self-efficacy for physical activity and a greater inclination toward sports participation (43).

The current work has additionally uncovered a negative correlation linking physical activity and internet addiction, which can significantly negatively predict internet addiction. This discovery was consistent with previous research findings (7, 44, 45). For instance, Alshehri et al. (45) suggested that students with higher levels of internet addiction reported less physical activity and higher weight based on changes in weight rate. Vandelanotte et al. (7) pointed out that the widespread utilization of the internet and computers might stand in inverse proportion to physical activities, as the time involved could otherwise be spent on physical activity. Moreover, Alaca et al. (44) posited that prolonged internet use could potentially give rise to musculoskeletal problems, thereby culminating in decreased levels of physical activity. Similarly, Barkley and Lepp (46) posited that spending more time online can be regarded as a sedentary behavior, leading to poor physical activity. Consistent with the discoveries of Senol-Durak et al. (21), our study also discerned a significant negative correlation between subjective well-being and internet addiction, meaning that the lower an individual’s subjective well-being, the higher their tendency toward internet addiction. Drawing from prior inquiries, subjective well-being is a crucial psychological factor that reduces risky behaviors (such as alcohol consumption, substance abuse, and internet addiction) and enhances self-control ability. More concretely, individuals with elevated subjective well-being demonstrate superior psychological health conditions, stronger self-control capabilities, and an increased proclivity to govern undesirable conduct (47). However, when confronted with the enticements of the internet, diminished subjective well-being may lead individuals toward adverse behaviors such as excessive internet usage or internet addiction. Furthermore, this finding aligned with the psychological need network satisfaction dominance theory posited by Deng et al. (48). This theory posits that subjective well-being serves as an external manifestation of an individual’s fulfilled psychological needs. Individuals, guided by the demands of subjective well-being, construct self-awareness, self-regulation, and self-control. Should issues arise during the establishment of self-awareness and the demands of subjective well-being be unsatisfied, individuals might resort to compensatory strategies, such as immersing themselves in easily gratifying pursuits like internet activities (48).

The present study indicated a significant positive correlation between physical activity and subjective well-being. This finding was consistent with the results of Lin et al. (49), to some extent suggesting that physical activity is a crucial protective factor for increasing happiness. The reasons why physical activity contributes to enhancing individual subjective well-being can be explained from several perspectives. Firstly, from a physiological perspective, studies revealed that routine physical activity stimulates neurotransmitter secretion, inducing a state of relaxed pleasure (50). Secondly, research indicated that engagement in physical activities was predominantly a communal endeavor. Throughout sports activities, it not only significantly enhances individuals’ social interactions but also fulfills their spiritual communication needs in social life. This, in turn, enables effective recognition of one’s own value by others, leading to enhanced satisfaction with social life (51). Similarly, it has also been shown that the positive impact of physical activity on individual subjective well-being is mediated through mental health, such as reducing negative emotions associated with subjective well-being or promoting the fulfillment of psychological needs and psychological resilience (52). Consequently, we posited that behaviors aimed at mitigating negative emotions or fulfilling psychological needs may bolster an individual’s subjective well-being (53). In addition to physical activity, various methods can be employed to boost subjective well-being, including parental or social support (54, 55), maintaining healthy sleep patterns (56), and avoiding a range of health-risk behaviors (47).

The results of the mediation analysis suggested that subjective well-being mediates the relationship between physical activity and internet addiction. This implied that physical activity not only directly influences the individual’s internet addiction, but when considering subjective well-being, this influence was predominantly manifested through the mediating role of subjective well-being, further impacting the internet addiction. The mediation process unfolded in two steps: initially, as individual physical activity levels rise, their perceived subjective well-being naturally increases; subsequently, with the strengthening of individual subjective well-being, the likelihood of excessive internet use or addiction may decrease. The initial step can be demonstrated and elucidated by the fact that engaging in physical activity can meet an individual’s fundamental psychological needs, consequently indirectly fostering an increase in well-being (50). The second step can be elucidated by the dominance theory of psychological needs and network satisfaction (48).

Besides the two basic processes of the mediating effect, the overall mediating results are also worthy of attention. Studies have shown that physical activity promotes the release, synthesis, and metabolism of dopamine in the body, eliciting a sense of pleasure and satisfaction akin to internet use and effectively mitigating the positive reinforcement from addictive sources (57). Additionally, physical activity fosters the neurogenesis, survival, and differentiation of neural stem cells or progenitor cells in the hippocampal dentate gyrus, enhancing the plasticity of hippocampal neural synapses and positively influencing individual cognitive functions (58). Nevertheless, compromised cognitive function usually results in emotional distress and impairment across multiple functional domains, including depression (15), anxiety (16), and negative peer relationships (59), all established as risk factors for internet addiction. Thus, engaging in physical activity could potentially enhance individual cognitive functions, enhance the experience of happiness, and consequently lower the occurrence of internet addiction. Therefore, improving subjective well-being is unquestionably an effective and practical regulatory strategy for coping with internet addiction among college students.

Undoubtedly, aside from the mediation of subjective well-being, the efficacy of physical activity in mitigating internet addiction is noteworthy. Firstly, from a neurobiological perspective, physical activity can foster adaptive remodeling of the reward circuit in individuals with internet addiction and bidirectionally regulate dopamine synthesis and release, thereby mitigating recurrent internet addictive behaviors (60). Secondly, as per Davis’s (61) cognitive-behavioral model, pathological internet use arises from distal factors like depression and life stress, coupled with maladaptive cognitive adaptations at the proximal level. Nevertheless, existing studies have demonstrated that physical activity enhances hippocampal cognitive function, facilitates the release of neurotrophic factors and endogenous substances, induces a relaxed and pleasurable mood, and holds potential anti-inflammatory and antidepressant effects (62). Additionally, early studies suggested that reducing psychological cravings and ameliorating attentional bias in addictive cues is fundamental to quitting the internet addiction (60). However, substance addiction research has found that physical activity can enhance top-down inhibitory control from the prefrontal cortex, accurately identify addiction-related cues, shift addicts’ attention, reduce cognitive resource consumption, and thereby lower psychological cravings and attentional biases toward relevant cues (63).

### Limitations and future directions

It should be admitted that this work has some limitations that need to be considered in future research. Firstly, due to the cross-sectional design adopted in this study, it was not possible to derive a causal relationship between the variables, and future research could employ longitudinal designs to explore more profound causal relationships between variables. Secondly, the reliance on self-reported data from college students in this study introduces the potential for influence from social support and recall bias. Given that, alternative methods, such as experimental research, can be employed to obtain more accurate and reliable data (64). Thirdly, the data for this study had to be collected online due to social restrictions during the COVID-19 period, leading to the exclusion of college students without internet access, and future research could utilize a mixed-methods approach, integrating online surveys, face-to-face interviews, or alternative data collection methods, to acquire a more varied and representative sample. Furthermore, diverse sampling strategies covering various disciplines, academic years, and geographical locations could be implemented to enhance the external validity of research outcomes. Moreover, this study employed convenient sampling, resulting in a moderately sized sample. Hence, future studies should contemplate opting for probability sampling and a larger sample size (65). Finally, beyond subjective well-being, future research should explore potential variables related to physical activity and internet addiction as mediating factors to elucidate the underlying mechanisms between them.

### Implications

Despite these limitations, the present study has also made several distinctive original contributions. Firstly, prior research predominantly concentrated on the isolated impact of internet addiction on either physical activity or subjective well-being, lacking a comprehensive exploration, especially during the COVID-19 period. Secondly, to the best of our knowledge, this study pioneers the investigation of the correlation between physical activity and internet addiction among Chinese college students amid the COVID-19 pandemic, concurrently examining the mediating role of subjective well-being. This significantly broadens the scope of internet addiction research. Illustrated by a straightforward mediation model, we effectively elucidate how physical activity ameliorates the internet addiction in college students and articulate practical strategies to mitigate this issue. Moreover, drawing on these findings, several crucial practical implications arise organically. Firstly, for a foundational approach to addressing the internet addiction in college students, parents and educators should provide standardized media literacy education to curtail unnecessary internet usage. Specifically, parents can instruct students on how to use the internet sensibly and emphasize the risks and potential harm of excessive internet use. Additionally, educators should nurture critical skills in students, empowering them to assess online content critically and make informed decisions. Secondly, as the crucial mediating function of subjective well-being between physical activity and internet addiction, parents and educators should implement measures to enhance students’ subjective well-being, including psychological counseling, emotional support, and positive interactions (66). Establishing a positive and supportive atmosphere in both home and educational environments contributes to increased overall happiness among students, acting as a buffer against the potential negative effects of internet addiction. Moreover, given the favorable impact of physical activity on alleviating internet addiction, students themselves should actively participate in extracurricular sports activities and consciously develop a routine of active participation in sports. Apart from that, parents and educators should actively promote and facilitate students’ engagement in extracurricular sports. Schools can introduce a variety of sports programs, and parents can endorse their children’s involvement in sports clubs or teams. This not only contributes to fostering physical well-being but also offers a positive means to alleviate daily academic pressures and boost happiness. Lastly, in line with earlier research, it is advisable for physical education teachers to incorporate more motivating learning tasks into regular physical education courses, fostering students’ positive motivation for adopting healthy behaviors (67), so as to elevate their physical activity levels and alleviate issues related to internet addiction.

## Conclusion

The present study identified notable gender differences in both physical activity and internet addiction, and active involvement in physical activities could diminish internet addiction among college students. More importantly, subjective well-being served as a mediating factor in the association between physical activity and internet addiction, suggesting that beyond participating in physical activities, enhancing subjective well-being proves to be an effective strategy for college students dealing with internet addiction. These findings are vital for devising focused intervention strategies and support plans within university settings. Recognizing the nuanced influence of gender on susceptibility to physical activity and internet addiction enables more targeted and efficient strategies. Moreover, interventions addressing psychological well-being would make a substantial contribution to mitigating internet addiction, offering a comprehensive approach to tackling this problem. In conclusion, our study not only offers valuable insights into comprehending gender disparities linked to physical activity and internet addiction but also underscores the crucial role of subjective well-being in mediating this intricate association. The outcomes of these studies aid in developing comprehensive strategies to tackle the broader issue of internet addiction among college students in China or even across the world.

## Data availability statement

The original contributions presented in the study are included in the article/supplementary material, further inquiries can be directed to the corresponding authors.

## Ethics statement

The studies involving humans were approved by the Research Ethics Committee at South China University of Technology. The studies were conducted in accordance with the local legislation and institutional requirements. The participants provided their written informed consent to participate in this study.

## Author contributions

JW: Conceptualization, Data curation, Formal analysis, Investigation, Methodology, Project administration, Software, Supervision, Writing – original draft, Writing – review & editing. XX: Conceptualization, Methodology, Supervision, Writing – review & editing. QW: Data curation, Formal analysis, Investigation, Writing – review & editing. CZ: Data curation, Formal analysis, Investigation, Methodology, Writing – review & editing. GY: Conceptualization, Funding acquisition, Investigation, Methodology, Project administration, Writing – review & editing.

## Footnotes

^1^https://www.wjx.cn/
